# Cross-Sectional Association of Urinary Bisphenol A and Vaccine-Induced Immunity against Hepatitis B Virus: Data from the 2003–2014 National Health and Nutrition Examination Survey

**DOI:** 10.3390/ijerph19031103

**Published:** 2022-01-19

**Authors:** Jun Young Uhm, Hyoung-Ryoul Kim

**Affiliations:** 1 Department of Medicine, Graduate School, The Catholic University of Korea, Seoul 06591, Korea; celeblue@naver.com; 2 Department of Occupational & Environmental Medicine, College of Medicine, The Catholic University of Korea, Seoul 06591, Korea

**Keywords:** BPA, vaccine-induced immunity, HBV, NHANES, immune response, cross-sectional analysis

## Abstract

Hepatitis B virus (HBV) infection poses a serious health burden; bisphenol A (BPA), a commonly used plasticizer for consumer products, is a potential immune disruptor. However, epidemiologic studies revealing the association between BPA exposure and immunity are limited. This study investigates the association between environmental BPA exposure and immune response following HBV vaccination in a nationally representative sample population. Using National Health and Nutrition Examination Survey data from six cycles, we analyzed the data of 6134 participants, classified as susceptible to HBV infection (*n* = 3086) or as having vaccine-induced immunity (*n* = 3048). Associations between BPA level and HBV susceptibility were assessed using multivariable logistic regression and expressed as odds ratios (ORs) of the pooled data and data for each cycle. There was a significant association in the pooled data after adjusting for potential confounders (adjusted OR (aOR): 1.14, 95% confidence interval (CI): 1.05–1.23). However, the associations between BPA concentration and HBV susceptibility were inconsistent across the survey cycles and tended to decrease in more recent cycles. Although this study preliminarily suggests that BPA attenuates the immune response to hepatitis B vaccination, further prospective studies are warranted to elucidate the discrepancies observed.

## 1. Introduction

Bisphenol A (BPA; CAS 80-05-7; IUPAC name: 4,4′-(propane-2,2-diyl)diphenol)), a phenolic compound with the chemical formula C_15_H_16_O_2_, is a plasticizer mainly used for manufacture of polycarbonate plastics or epoxy resins. As one of the most widely produced chemicals worldwide, BPA is found in various items, such as plastic containers, food packaging materials, toys, office supplies, and dental sealants; thus, BPA exposure can occur via multiple sources [1,2].

When BPA is absorbed into the human body, it binds to several hormone receptors, including the estrogen receptor (ER) [3,4,5]. As an endocrine-disrupting chemical, BPA is known to increase the risk of reproductive toxicity, developmental disorders, immunotoxicity, and several endocrine diseases, such as diabetes, breast cancer, and obesity [6].

Approximately 257 million individuals worldwide are infected with the hepatitis B virus (HBV) [7], which can progress to liver cirrhosis or liver cancer in approximately 15–40% of the carriers if not treated [8]; hence, it remains a serious public health concern, as HBV is a major cause of liver cancer, which accounts for the third highest cancer deaths [9,10].

Hepatitis B vaccination is recommended as a preventive measure; however, in some cases, sufficient immunity against HBV cannot be produced as antibodies are not generated because of a decline in immune response even after vaccination [11]. Several studies have provided direct and indirect evidence that BPA modulates the immune response [12,13]. In particular, it has been reported that exposure to BPA causes apoptosis, or necrosis, of immune cells [14,15], decreased antigen presentation [16] and suppression of adaptive immunity [17,18], which can ultimately decline overall immunity. Since antibody formation post vaccination is achieved via a series of both innate and adaptive immune responses [19], exposure to BPA may decrease antibody formation following HBV vaccination.

However, the effect of BPA on the immune response following hepatitis B vaccination has not yet been investigated. Thus, in this study, we investigate the association between BPA exposure and the immune response following hepatitis B vaccination using nationally representative National Health and Nutrition Examination Survey (NHANES) data.

## 2. Materials and Methods

### 2.1. Study Population

**Figure 1:**
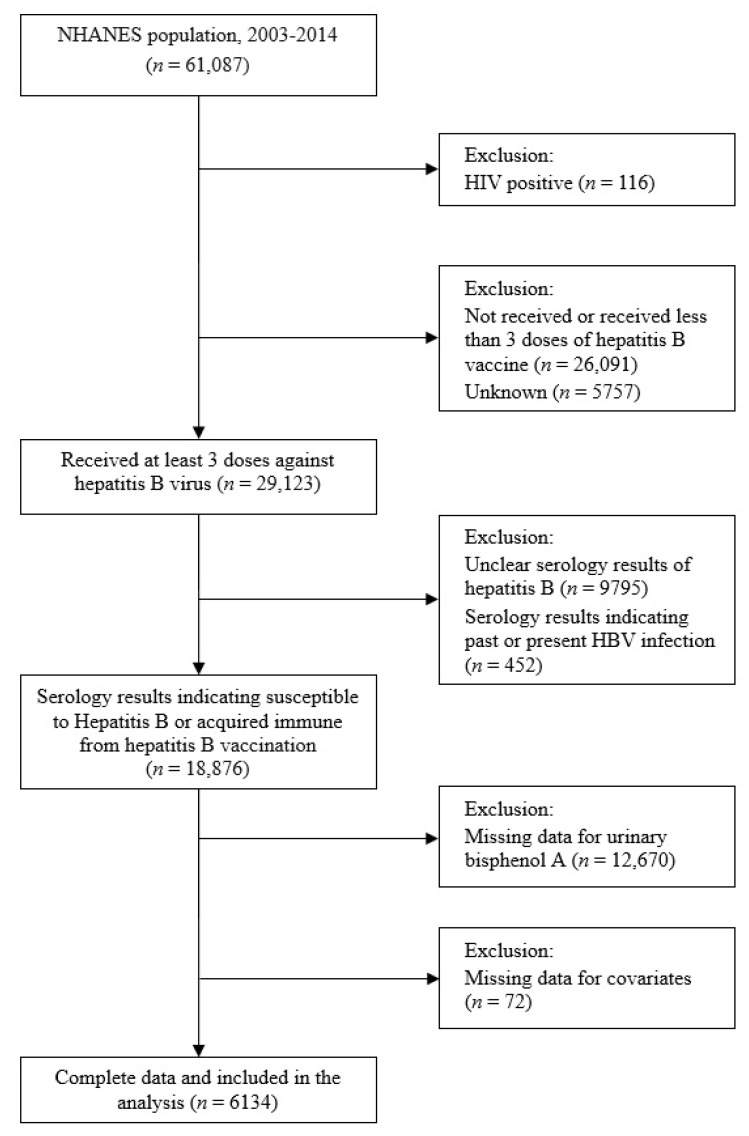
Flow chart illustrating eligibility of the study participants. NHANES: National Health and Nutritional Examination Survey (2003–2014).

Data from six cycles (2003–2004, 2005–2006, 2007–2008, 2009–2010, 2011–2012, and 2013–2014) of the NHANES, a stratified random sample survey designed to be representative of the US population, were pooled. The selection of study participants is summarized in Figure 1. Of the 61,087 NHANES respondents, we excluded the following: those who tested positive for HIV (*n* = 116) and were likely to be immunocompromised, those who had not received at least three doses of hepatitis B vaccine (*n* = 26,091), and those whose vaccination status was unknown (*n* = 5757). We also excluded those with unclear serologic results for hepatitis B (*n* = 9795) or those with serologic results suggestive of past or present HBV infection (*n* = 452). Finally, after excluding those with no BPA concentration data (*n* = 12,670) or with missing potential covariate data (*n* = 72), the data of 6134 participants were analyzed.

### 2.2. Ethics Statement

Informed consent was obtained from all participants of the 2003–2014 NHANES, and the study protocol was approved by the Ethics Review Committee of the National Center for Health Statistics under the Centers for Disease Control and Prevention (CDC) prior to carrying out the research [20]. Because NHANES is a publicly available dataset, this study was deemed exempt from review by the institutional review board of the Catholic University of Korea.

### 2.3. Exposure Variable

Urinary BPA concentrations of single spot urine samples collected from one-third of a subset of randomly selected participants from each NHANES cycle were measured by the Division of Environmental Health Laboratory Sciences (National Center for Environmental Health, CDC). For accurate quantification, the conjugated form was converted to free BPA by hydrolysis before subjecting to on-line solid-phase extraction isotope dilution high-performance liquid chromatography-tandem mass spectrometry.

The lower limit of detection (LLOD) of urinary BPA was 0.36, 0.4, and 0.2 ng/mL from 2003–2004, 2005–2012, and 2013–2014, respectively. The respective ratio of the LLOD to the number of participants in each cycle was 6.5%, 7.1%, 6.3%, 8.0%, 10.3%, and 4.0% in 2003–2004, 2005–2006, 2007–2008, 2009–2010, 2011–2012, and 2013–2014. If urinary BPA concentration < LLOD, the LLOD was substituted with the value divided by the square root of 2 [21]. Because the LLOD values for 2003–2004 and 2012–2014 were smaller than those for 2005–2011, the LLOD (0.4 ng/mL) in 2005–2011 divided by the square root of 2 (0.28) was applied as the LLOD in 2003–2004 and 2012–2014 to match the LLOD in all cycles as previously described [22,23].

### 2.4. Outcome Variables

Serum tests for the HBV were performed in the CDC’s Division of Viral Hepatitis after intravenous blood samples were collected from participants aged ≥ 6 years. The hepatitis B surface antibody (anti-HBs) level was measured using the solid-phase competitive enzyme immunoassay (Ausab, Abbott Laboratories), and the hepatitis B core antibody (anti-HBc) level was measured using the quantitative enzyme-linked immunosorbent assay (Vitros, anti-HBc ELISA). When the sample tested positive for anti-HBc, a test for the hepatitis B surface antigen (HBsAg) (Auszyme, Abbott Laboratories) was additionally performed. Otherwise, when the sample tested negative for anti-HBc, HBsAg status was also considered negative.

These three serological indicators were recorded qualitatively (positive or negative) and indicated the immune status against the HBV. When a sample tested negative for anti-HBs, anti-HBc, and HBsAg, it was classified as “susceptible to HBV.” When it tested negative for only HBsAg, it was classified as “past HBV infection.” When it tested positive for anti-HBs and negative for both anti-HBc and HBsAg, it was classified as “vaccine-induced immunity to HBV infection.” Lastly, when it tested negative for only anti-HBs, it was classified as “present HBV infection” [24,25]. As those with serum results suggesting past or current HBV infection were excluded from this study, participants were mainly divided into “susceptible to HBV” group (*n* = 3086) and “vaccine-induced immunity to HBV infection” group (*n* = 3048).

### 2.5. Covariates

As a priori literature review revealed that immune response to HBV vaccine was independently associated with age, sex, country of birth, smoking status, and body mass index (BMI) [11,26,27], and that BPA concentration was independently associated with age, sex, race/ethnicity, household income, and smoking status [28,29,30], these variables were selected as covariates. Age, sex, race/ethnicity, country of birth, and family income data were collected based on the information filled out in the self-report questionnaire. Prior to the analysis, the study participants were stratified by age into the following groups: 6–19, 20–39, and ≥40 years. They were also stratified by sex (male or female). Regarding race/ethnicity, the participants were classified as Non-Hispanic White, Non-Hispanic Black, Hispanic, or Other race/Multi-racial. Based on the birth country, they were classified as “born in the US” or “born elsewhere.” Participants were stratified by family income based on the family-income-to-poverty ratio (FIPR), with FIPR ≤ 1.3 as low income, 1.3 < FIPR < 3.5 as middle income, and FIPR > 3.5 as high income [31]. Smoking status was classified based on the serum cotinine concentration, with a serum cotinine concentration <10 ng/mL indicating nonsmoker status, and ≥10 ng/mL indicating smoker status [32].

BMI was calculated by dividing the measured weight (kg) by the square of the measured height (m^2^), and adults aged > 20 years were classified into the following categories based on BMI: underweight (<18.5), normal weight (18.5–24.9), overweight (25–29.9), or obese (≥30). Participants aged < 20 years were classified based on the CDC growth chart for cut-offs by age and sex, with those in the top 5% as obese, those in the top 5–15% as overweight, those in the top 15–95% as normal weight, and those in the bottom 5% as underweight [33,34]. Urinary creatinine concentration was initially measured using the Jaffe reaction (Beckman CX3) method; however, the enzymatic method (Roche ModP), an improved method, was introduced in 2007 to address potential interference associated with the Jaffe reaction. Therefore, to compare the urinary creatinine concentration measured by different methods before and after 2007, data from 2003–2006 were adjusted based on the following equations according to the NHANES analysis guidelines [35]:

If urinary creatinine level < 75, *Y* = [1.02 × √(*X*) − 0.36]^2^.

If 75 < urinary creatinine level < 250, *Y* = [1.05 × √(*X*) − 0.74]^2^.

If urinary creatinine level ≥ 250, *Y* = [1.01 × √(*X*) − 0.10]^2^.

where *Y* is the adjusted creatinine level (mg/dL), and *X* is the unadjusted creatinine level (mg/dL).

### 2.6. Statistical Analysis

SAS statistical software (version 9.4; SAS Institute Inc.; Cary, NC, USA) was used to perform all statistical analyses. Statistical significance was set at *p* < 0.05 (two-tailed test). Descriptive statistical analysis was used, considering all primary sampling units, stratification variables (strata), and environmental sampling weights of the six cycles. Categorical variables are presented as an unweighted count and a weighted percentage, and trends over time were analyzed using univariate logistic regression with the survey cycle as an independent variable. As urinary creatinine and urinary BPA concentrations, which are continuous variables, exhibited a right-skewed distribution, they are presented as the weighted arithmetic mean and standard error after transformation into natural logarithms for normalization, and trends over time were analyzed using univariate linear regression with the survey cycle as an independent variable.

We determined whether there were significant differences between the two groups according to anti-HBs positivity after the administration of at least three doses of HBV vaccination. Categorical variables are presented as the unweighted number of participants and weighted percentages, and differences between groups were analyzed using the Rao–Scott chi-squared test. Continuous variables are presented as the weighted arithmetic mean and standard error, and the differences between groups were analyzed by Student’s *t*-test.

To analyze the association between the urinary BPA concentration and the prevalence of HBV susceptibility, the odds ratios (ORs) and 95% confidence interval (CI) for the pooled data and those from each cycle were calculated using multivariate logistic regression. Then, the trends of ORs over cycle were analyzed. The analysis model was established by adjusting the covariates selected a priori as follows.

Model 1: Natural log-transformed urinary creatinine was adjusted. (For pooled data, the survey cycle was additionally adjusted).

Model 2: Age and sex were adjusted, in addition to the covariates included in Model 1.

Model 3: Race/ethnicity, country of birth, family income, smoking status, and BMI were adjusted, in addition to the covariates included in Model 2.

To determine whether the logistic regression model had a good fit with the relationship between the natural log-transformed BPA (ln(uBPA)) and the log-odds of “susceptible to HBV” satisfying linearity, we performed restricted cubic spline (RCS) regression analysis with four degrees of freedom (knots at 10th, 50th, and 90th percentiles) by adjusting the covariates included in Model 3 using SAS Macro (%RCS_Reg, V1.50) [36]. As the log-odds of “susceptible to HBV” increased linearly according to the ln(uBPA) concentration in the spline curve (*p* for non-linearity = 0.87) (Figure S1), the ln(uBPA) concentration was designated as an exposure variable in the final regression model. The shape of the dose–response curve of “susceptible to HBV” according to the urinary BPA concentration was also examined using the RCS graph. Finally, subgroup analysis was performed to examine the effect modification of the association between urinary BPA and “susceptibility to HBV” by age, sex, race/ethnicity, country of birth, family income, smoking status, and BMI.

## 3. Results

Table 1 shows the general characteristics of the study participants based on the pooled data and the data for each cycle. Variables, such as age, race/ethnicity, household income, BMI, and urinary BPA concentration, showed significant trends in each cycle. In terms of age, participants aged 6–19 years showed a decreasing trend (*p* < 0.001), whereas those aged ≥ 40 years showed an increasing trend (*p* = 0.035). Other race/multi-racial groups showed an increasing trend (*p* = 0.006). Regarding family income, the low-income group showed an increasing trend (*p* < 0.001), whereas the high-income group showed a decreasing trend (*p* = 0.008). The normal BMI group showed a significantly decreasing trend (*p* = 0.018), and urinary BPA concentration also showed a decreasing trend (*p* < 0.001). However, sex, country of birth, smoking status, and urinary creatinine level did not show significant trends in any cycle (Table 1).

Urinary BPA concentration was significantly higher in the “susceptible to HBV” group than in the “vaccine-induced immunity to HBV infection” group (*p* = 0.007). In terms of age and sex, the “vaccine-induced immunity to HBV infection” group showed a significantly lower age distribution than the “susceptible to HBV” group (*p* < 0.001), with a higher proportion of women (*p* = 0.004). The proportions of Non-Hispanic White and Other race participants were significantly higher in the “vaccine-induced immunity to HBV infection” group, whereas those of Non-Hispanic Black and Hispanic participants were significantly higher in the “susceptible to HBV” group (*p* < 0.001). Family income was significantly higher in the “vaccine-induced immunity to HBV infection” group (*p* < 0.001). The proportions of obese participants and smokers were significantly higher in the “susceptible to HBV” group (*p* < 0.001 and *p* = 0.002, respectively). There was no significant difference between the two groups in terms of country of birth and urinary creatinine level (Table 2).

Table 3 shows the association between the urine BPA concentration and the prevalence of HBV susceptibility based on the pooled data for all cycles and the data for each cycle. After adjusting for all covariates included in model 3, the association in the pooled data was significant (adjusted OR (aOR): 1.14, 95% confidence interval (CI): 1.05–1.23). As for the individual cycles, significant association was observed in three consecutive cycles from 2003–2004 to 2007–2008 (aOR: 1.35, 95% CI: 1.13–1.61; aOR: 1.43, 95% CI: 1.16–1.78; and aOR: 1.27, 95% CI: 1.03–1.57 in NHANES 2003–2004, 2005–2006, and 2007–2008 cycles, respectively); however, no significant association was observed in the remaining three cycles from 2009–2010 to 2013–2014 (aOR: 1.11, 95% CI: 0.96–1.30; aOR: 1.13, 95% CI: 0.92–1.39; and aOR: 0.87, 95% CI: 0.70–1.07 in NHANES 2009–2010, 2011–2012, and 2013–2014 cycles, respectively). Reduced association was observed in more recent cycles (*p*-trend = 0.028 in model 1, *p*-trend = 0.017 in model 2, and *p*-trend = 0.036 in model 3) (Table 3).

**Figure 2:**
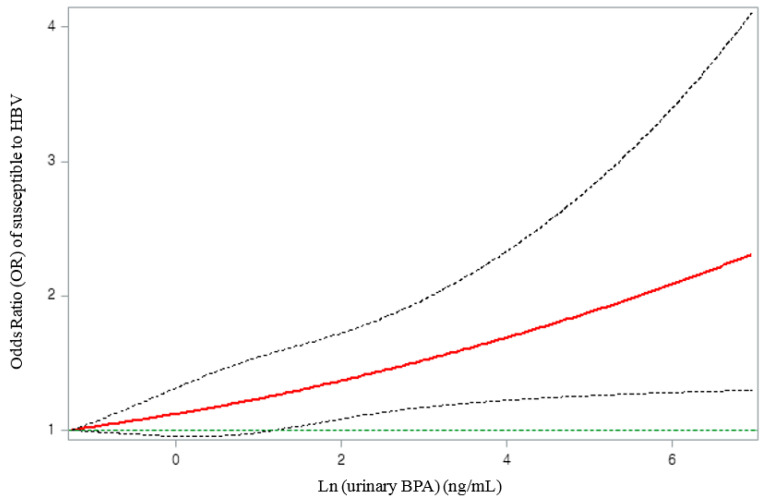
Associations of immune response to vaccination against HBV with natural log-transformed urinary BPA concentration, adjusted for natural log-transformed urinary creatinine, survey cycle, age, sex, race/ethnicity, country of birth, household income, body mass index, and smoking status. The solid line represents the smoothing trends estimated from the restricted cubic spline with four degrees of freedom (knots at 10th, 50th, and 90th percentiles), and the dashed lines represent its 95% confidence intervals (CIs). The green horizontal dashed line represents the reference odds ratio (1.0).

The spline curve of the dose–response relationship of the “susceptible to HBV” group according to the urinary BPA concentration is shown in Figure 2. A monotonic increase in the OR for “susceptible to HBV” was observed with an increase in the urinary BPA concentration (Figure 2).

According to the subgroup analysis results, no effect modification was noted for age, sex, race, family income, smoking status, and BMI (Figure S2).

## 4. Discussion

Using a large multi-racial sample representative of the US population, this study investigated the association between urinary BPA concentrations and immune responses after HBV vaccination. We observed an increase in the odds for susceptibility to HBV infection with an increase in urinary BPA concentration in the pooled analysis, which suggests that an increase in urinary BPA concentration is associated with a decrease in the effectiveness of HBV vaccination. In other words, exposure to BPA may induce susceptibility to hepatitis B even after vaccination; hence reduction of products made of BPA may contribute to the prevention of the spread of hepatitis B.

Immunomodulation induced by BPA exposure involves complex cell signaling pathways initiated by the binding of BPA with nuclear receptor families [12]. Thus, various effects related to immunity have been reported depending on the model organisms and the BPA concentrations [13]. Although data regarding the mechanism underlying vaccine-induced immune response remains limited, several studies have proposed possible mechanisms, as follows:

First, dendritic cells are representative antigen-presenting cells that mediate innate and adaptive immunity [37] and induce differentiation of naïve CD4^+^ T lymphocytes into helper T lymphocytes by presenting HBsAg in the early stage of the immune response to the HBV vaccine [19]. These dendritic cells are differentiated from monocytes [38]. A previous study has reported that apoptosis and necrosis of human monocytes increase with an increase in the BPA concentration, resulting in decreased cell viability [14]. Thus, an increase in the BPA concentration may decrease the number of monocytes and a consequent decrease in that of dendritic cells. Moreover, antigen presentation must be preceded by antigen capture through the endocytosis of dendritic cells [39]. An in vitro study showed that exposure to BPA decreases the endocytic capacity of human dendritic cells [16]. Therefore, BPA is expected to reduce antigen capture in dendritic cells, ultimately reducing the efficiency of antigen presentation of the HBV vaccine antigen.

In terms of adaptive immunity, the immunoglobulin G (IgG) subclass of anti-HBs generated after HBV vaccination is mainly type 1 (IgG1) [40,41,42]. Both type 1 and 2 helper T lymphocytes (Th1 and Th2, respectively) are involved in the production of IgG1 [43,44,45]. In line with this theory, some studies have revealed that high responders to the HBV vaccine have significantly higher levels of Th1 cytokines, Il-2 and IFN-γ, and Th2 cytokines, Il-10 and Il-13, than did non-responders [46,47]. A recent study on participants vaccinated with HBV vaccines also reported that the levels of IFN-γ, a Th1 cytokine, and Il-13, a Th2 cytokine, are positively correlated with the anti-HBs titer [48]. Hence, the decrease in the levels of IFN-γ [17], Il-10, and Il-13 [18] observed upon the BPA treatment of human peripheral blood mononuclear cells indicate that BPA suppresses both Th1- and Th2-type immune responses for antibody formation, resulting in low anti-HBs titers.

Finally, a study on human B lymphocytes reported that BPA exhibited cytotoxic effects on B lymphocytes by generating reactive oxygen species (ROS) in the body [15]. As the number of HBsAg-specific memory B lymphocytes reflects the immune response to the HBV vaccine [49], a decrease in the number of HBsAg-specific memory B lymphocytes induced by ROS production due to BPA may also reduce the immune response to the vaccine. In summary, BPA may reduce the effectiveness of hepatitis B vaccination by suppressing the immune response.

Regarding the trends of the variables according to each cycle, urinary BPA concentration decreased over time, whereas the prevalence of HBV susceptibility increased. This contradicts our hypothesis that BPA inhibits the immune response to vaccines. Such results can be partially explained by previous findings of a decrease in immunity from the HBV vaccine in NHANES cohorts of children and adolescents registered to be born between 1994 and 2003, despite an increase in HBV vaccine coverage over time [50]. The prevalence of HBV susceptibility status might have increased over the course of this study due to the increase in the proportion of study participants born between 1994 and 2003 in the more recent NHANES data. In addition, as aging negatively affects the immune response to the HBV vaccine [11,27], and as the mean age of participants tends to increase over time in this study, the participants’ overall immune response to the HBV vaccine may have been lower in the more recent survey cycles.

For the association between the BPA level and immune response to the HBV vaccine in each cycle, it was only significant in three cycles (2003–2004, 2005–2006, and 2007–2008), and the association tended to decrease in more recent cycles. Although not evident, there are some plausible explanations for these inconsistencies.

First, in the subgroup analysis, effect modification was not observed for all the covariates, including age, race, economic status, and BMI, which tended to show a significant tendency by cycle. This suggests that the change in the OR was not caused by the changes in the covariates in each cycle. However, the inconsistencies may be due to proportional differences of unidentified effect modifiers across the survey cycles.

Second, as the harmful effects of BPA have been revealed in several studies, since the 2000s, several campaigns and policies to reduce the use of BPA have emerged [51,52]. Accordingly, the urinary BPA concentration, which reflects the exposure to BPA, showed a decreasing trend over time. This has also been reported in studies investigating the temporal trend of BPA exposure [53]. BPA can induce changes in the immune response by binding to the ER on the surface of immune cells [12]. An in vitro study also revealed detectable estrogenic activity of BPA, which increased in a concentration-dependent manner [54]. Therefore, when the concentration of BPA decreases, the estrogenic activity in the immune cells is predicted to decrease, with the negative effect on immunity almost lost when the concentration reaches the threshold. Interestingly, similar results were reported by a study investigating the association between BPA exposure and pediatric obesity for each survey cycle using the same 2003–2014 NHANES data used in this study. A significant correlation was identified until 2008; however, no significant value was identified in subsequent cycles [55]. Furthermore, a study using the 2003–2008 NHANES data reported a significant association between BPA exposure and type 2 diabetes in the 2003–2004 cycle; however, no significant association was noted in the two cycles after 2005 [23]. In both studies, the urinary BPA concentration was significantly higher in the cycles with a significant association than in those with no significant association, similar to our findings.

Similar to some studies with almost identical participants and study designs except for the independent variables [56,57], this study performed logistic regression analysis to examine the association between the urinary BPA concentration and the immune response after hepatitis B vaccination. Although it is common to use logistic regression to analyze dichotomous outcome variables in a cross-sectional analysis, the OR obtained using logistic regression is overestimated when the outcome of the study groups is common [58]. However, because the assumption of homogeneity is more tenable with the OR than with the risk ratio (RR), previous have studies recommend using OR as an effect measure even if the outcome is common, as long as careful attention is paid to the interpretation of results [59,60]. Several studies have been based on such recommendation [61,62,63]. Therefore, when interpreting the OR as an effect measure, the study results will be valid as long as care is taken to avoid interpreting the ORs as RRs.

To the best of our knowledge, this is the first study to investigate the association between urinary BPA concentration and immune function after vaccination, as well as the change in this association over time. This study analyzed the 12-year NHANES data to examine the trend over time with larger sample size. By increasing the sample size, we were also able to incorporate various confounding variables identified in previous studies into the statistical model.

This study had several limitations. First, owing to the nature of the NHANES data, we used a cross-sectional study design. Because the data for the urine BPA concentration and the presence of antibodies after hepatitis B vaccination in the participants were collected at the same time, it was impossible to establish a causal relationship. Furthermore, since the NHANES data do not indicate the times of administrations of the three doses of HBV vaccines, some participants may be categorized as “susceptible to HBV” due to the decline in antibody titers over time, regardless of BPA exposure. Such cases, however, should be rare since the titer is known to maintain for more than 30 years after HBV vaccination [64]. Second, information on hepatitis B vaccination history was collected from self-reported responses, which could cause recall bias and misclassification. To reduce this bias, documented vaccine data should be used. Third, considering the possible fluctuations in BPA concentration, using single spot urine samples might have led to a measurement bias. Therefore, we suggest that spot urine samples should be collected several times to account for the factors that may affect urinary BPA concentration, such as diet and urination. Finally, BPA exists as free and conjugated BPA in vivo, and only free BPA has been shown to exhibit adverse biological effects by binding to the ER [65]. However, the data used in this study measured the urinary BPA concentration after the deconjugation of conjugated BPA into free BPA; thus, the urinary BPA concentration data might not have accurately reflected the correct urinary BPA concentration. Therefore, future studies should consider measuring the free BPA concentration.

## 5. Conclusions

Using data representing the US population, this study identified a negative correlation between the urinary BPA concentration and the immune response after hepatitis B vaccination. Furthermore, a decrease in this correlation over time was observed. Although this epidemiological study provides preliminary evidence that environmental exposure to BPA may contribute to reduced immunity to hepatitis B vaccination, further studies based on the prospective study design remain warranted to elucidate these discrepancies.

## Figures and Tables

**Table 1:** Characteristics of selected participants (*n* = 6134) by survey cycle from NHANES (2003–2014).

		NHANES Survey Cycle						*p*-Trend ^a^
Characteristics	Pooled	2003–2004	2005–2006	2007–2008	2009–2010	2011–2012	2013–2014	
N	6134	970	1075	948	1037	971	1133	
Age (years)								
6–19	3785 (41.9)	709 (47.9)	749 (44.0)	576 (45.3)	588 (40.7)	536 (38.1)	627 (37.8)	<0.001
20–39	1375 (33.5)	155 (30.7)	220 (34.9)	185 (28.4)	275 (35.3)	262 (34.4)	278 (36.3)	0.074
≥40	974 (24.5)	106 (21.4)	106 (21.1)	187 (26.3)	174 (24.0)	173 (27.5)	228 (25.9)	0.035
Sex								
Female	3216 (53.1)	507 (53.8)	559 (52.7)	488 (52.8)	548 (55.2)	511 (53.0)	603 (51.5)	0.539
Race/Ethnicity								
Non-Hispanic White	2089 (63.4)	316 (65.9)	366 (66.3)	334 (65.8)	395 (62.4)	307 (61.9)	371 (59.6)	0.169
Non-Hispanic Black	1587 (13.0)	326 (14.5)	306 (13.7)	231 (12.9)	204 (12.7)	246 (11.9)	274 (13.1)	0.532
Hispanic	1886 (15.9)	285 (13.6)	343 (14.0)	344 (15.8)	358 (16.3)	242 (17.3)	314 (17.8)	0.233
Other race/multi-racial	572 (7.6)	43 (6.0)	60 (6.0)	39 (5.5)	80 (8.6)	176 (8.9)	174 (9.5)	0.007
Country of Birth								
Born in the US	5261 (88.7)	865 (92.2)	933 (87.3)	812 (89.9)	894 (88.6)	789 (86.4)	968 (88.9)	0.223
Family-Income-to-Poverty Ratio								
Low (<1.3)	2682 (30.6)	377 (25.0)	392 (22.9)	406 (29.4)	479 (31.8)	467 (35.0)	561 (36.9)	<0.001
Middle (1.3–3.5)	2021 (33.9)	349 (35.0)	407 (38.1)	325 (32.3)	332 (33.7)	289 (32.5)	319 (32.3)	0.223
High (>3.5)	1431 (35.5)	244 (40.0)	276 (39.0)	217 (38.3)	226 (34.5)	215 (32.5)	253 (30.8)	0.008
BMI Categories (kg/m^2^) ^b^								
Underweight	157 (2.5)	19 (1.9)	31 (3.4)	26 (2.3)	21 (1.7)	35 (3.7)	25 (1.9)	0.986
Normal weight	2947 (44.4)	511 (47.7)	556 (47.8)	438 (46.2)	477 (43.1)	446 (42.6)	519 (40.4)	0.018
Overweight	1383 (25.4)	194 (23.3)	231 (23.6)	213 (23.9)	252 (28.5)	230 (27.0)	263 (25.6)	0.163
Obese	1647 (27.7)	246 (27.0)	257 (25.2)	271 (27.6)	287 (26.7)	260 (26.6)	326 (32.1)	0.080
Smoking Status ^c^								
Smoker	859 (18.0)	117 (16.8)	146 (19.3)	124 (17.4)	152 (17.0)	137 (17.7)	183 (19.1)	0.728
HBV Serology								
Susceptible to HBV	3086 (50.5)	381 (45.7)	433 (43.6)	490 (49.0)	549 (49.8)	541 (53.4)	692 (58.9)	<0.001
Ln (uCr) (mg/dL)	4.61 ± 0.01	4.64 ± 0.03	4.63 ± 0.04	4.66 ± 0.03	4.58 ± 0.03	4.51 ± 0.04	4.64 ± 0.03	0.153
Ln (uBPA) (μg/L)	0.66 ± 0.02	1.09 ±0.08	0.73 ±0.05	0.85 ±0.07	0.62 ± 0.04	0.48 ± 0.04	0.34 ±0.05	<0.001

Values are expressed as unweighted count and weighted percentage N (%) for categorical variables and as weighted mean ± standard error for continuous variables. ^a^ Calculated by survey-weighted logistic regression for each subgroup of categorical variables and survey-weighted linear regression for continuous variables. ^b^ BMI categories: underweight (<18.5 kg/m^2^), normal weight (18.5–24.9 kg/m^2^), overweight (25–29.9 kg/m^2^), and obese (≥30 kg/m^2^) for participants ≥ 20 years of age. BMI under the age of 20 was categorized based on Centers for Disease Control and Prevention (CDC) growth charts. ^c^ Smoking status: Nonsmoker (serum cotinine < 10 ng/mL) and smoker (serum cotinine ≥ 10 ng/mL). Abbreviations—BMI: body mass index; NHANES: National Health and Nutrition Examination Survey; HBV: hepatitis B virus; uBPA: urinary bisphenol A; uCr: urinary creatinine.

**Table 2:** Characteristics of selected participants (*n* = 6134) from 2003–2014 NHANES with serology indicating susceptible to HBV or acquired immunity from HBV vaccination.

Characteristics	Susceptible to HBV (*n* = 3086)	Immunity from HBV Vaccination (*n* = 3048)	*p*-Value ^a^
Age (years)			<0.001
6–19	1802 (41.5)	1983 (42.4)	
20–39	659 (29.6)	716 (37.5)	
≥40	625 (28.9)	349 (20.1)	
Sex			0.004
Male	1535 (49.5)	1383 (44.2)	
Female	1551 (50.5)	1665 (55.8)	
Race/Ethnicity			<0.001
Non-Hispanic White	1063 (62.1)	1026 (64.8)	
Non-Hispanic Black	792 (13.7)	795 (12.4)	
Hispanic	965 (17.6)	921 (14.2)	
Other race/multi-racial	266 (6.6)	306 (8.6)	
Country of Birth			0.813
Born in the US	2672 (88.9)	2589 (88.6)	
Born elsewhere	414 (11.1)	459 (11.4)	
Family Income Poverty Ratio			<0.001
Low (<1.3)	1432 (32.8)	1250 (28.3)	
Middle (1.3–3.5)	1012 (35.3)	1009 (32.4)	
High (>3.5)	642 (31.9)	789 (39.3)	
BMI Categories (kg/m^2^) ^b^			<0.001
Underweight	67 (2.3)	90 (2.7)	
Normal weight	1354 (40.2)	1593 (48.7)	
Overweight	717 (25.3)	666 (25.5)	
Obese	948 (32.1)	699 (23.1)	
Smoking Status ^c^			0.002
Nonsmoker	2610 (80.2)	2665 (84.0)	
Smoker	476 (19.8)	383 (16.0)	
Ln (uCr) (mg/dL)	4.63 ± 0.02	4.59 ± 0.02	0.078
Ln (uBPA) (μg/L)	0.71 ± 0.03	0.61 ± 0.03	0.007

Values are expressed as unweighted count and weighted percentage N (%) for categorical variables and as weighted mean ± standard error for continuous variables. ^a^ calculated by Rao–Scott chi-squared test for categorical variables and Student’s t-test for continuous variables. ^b^ BMI categories: underweight (<18.5 kg/m^2^), normal weight (18.5–24.9 kg/m^2^), overweight (25–29.9 kg/m^2^), and obese (≥30 kg/m^2^) for participants ≥ 20 years of age. BMI under the age of 20 was categorized based on Centers for Disease Control and Prevention (CDC) growth charts. ^c^ Smoking status: nonsmoker (serum cotinine < 10 ng/mL) and smoker (serum cotinine ≥ 10 ng/mL). Abbreviations—BMI: body mass index; NHANES: National Health and Nutrition Examination Survey; HBV: hepatitis B virus; uBPA: urinary bisphenol A; uCr: urinary creatinine.

**Table 3:** ORs of the association between Ln(uBPA) levels and serology indicating not acquired immunity against HBV for NHANES participants who self-reported at least three doses of hepatitis B vaccination (*n* = 6134) (by cycle).

	Pooled	NHANES Survey Cycle						
		2003–2004	2005–2006	2007–2008	2009–2010	2011–2012	2013–2014	*p*-Trend
Model 1 ^a^	1.15 (1.06–1.25)	1.23 (1.05–1.45)	1.33 (1.05–1.69)	1.23 (1.04–1.46)	1.12 (0.95–1.32)	1.19 (0.95–1.49)	0.89 (0.73–1.08)	0.028
Model 2 ^b^	1.17 (1.08–1.27)	1.39 (1.19–1.63)	1.42 (1.13–1.78)	1.32 (1.10–1.59)	1.13 (0.97–1.32)	1.16 (0.95–1.40)	0.89 (0.73–1.09)	0.017
Model 3 ^c^	1.14 (1.05–1.24)	1.35 (1.13–1.61)	1.43 (1.16–1.78)	1.27 (1.03–1.57)	1.11 (0.96–1.30)	1.13 (0.92–1.39)	0.87 (0.70–1.07)	0.036

^a^ Model 1 is a logistic regression model adjusted for natural log-transformed urinary creatinine and survey cycle for pooled data. ^b^ Model 2 is a logistic regression model adjusted for age (6–19, 20–39, or >40), sex (male or female) in addition to model 1 covariates. ^c^ Model 3 is a logistic regression model adjusted for race/ethnicity (Mexican American, Other Hispanic, Non-Hispanic White, Non-Hispanic Black, or Other race/Multi-racial), country of birth (born in the US or elsewhere), family income poverty ratio (≤1.3, 1.3–3.5, or >3.5), BMI categories (underweight, normal weight, overweight, or obese), and smoking status (nonsmoker or smoker) in addition to model 2 covariates. Abbreviations: NHANES: National Health and Nutrition Examination Survey; uBPA: urinary Bisphenol A; HBV: hepatitis B virus; OR: odds ratio.

## Data Availability

All relevant data used in this study (2003–2014 NHANES) are freely available at https://www.cdc.gov/nchs/nhanes/index.htm (accessed on 30 October 2021).

## Supplementary Materials

The following are available online at https://www.mdpi.com/article/10.3390/ijerph19031103/s1, Figure S1. Log-odds of immune response to vaccination against hepatitis B virus according to natural log-transformed urinary bisphenol A, adjusted for natural log-transformed urinary creatinine, survey cycle, age, sex, race/ethnicity, country of birth, household income, body mass index, and smoking status. Figure S2. Subgroup analysis of odds ratio (OR) of susceptibility to HBV according to urinary BPA level. All covariates included in model 3 were adjusted.
