# Supplementary material for: Cross-Sectional Association of Urinary Bisphenol A and Vaccine-Induced Immunity against Hepatitis B Virus: Data from the 2003–2014 National Health and Nutrition Examination Survey
Source: Int J Environ Res Public Health. 2022 Jan 19;19(3):1103. doi: 10.3390/ijerph19031103 (PMC8834708; doi:10.3390/ijerph19031103)
Supplement: Supplementary file 1 [file ijerph-19-01103-s001.zip › ijerph-1481673-supplementary.pdf]

Supplementary Materials for

Cross-Sectional Association of Urinary Bisphenol A and Vaccine-Induced Immunity against Hepatitis B Virus: Data from the 2003–2014 National Health and Nutrition Examination Survey

Jun Young Uhm [1] and Hyoung-Ryoul Kim [1,2,*]

[1] Department of Medicine, Graduate School, The Catholic University of Korea, Seoul 06591, Korea; celeblue@naver.com
[2] Department of Occupational & Environmental Medicine, College of Medicine, The Catholic University of Korea, Seoul 06591, Korea
* Correspondence: cyclor@catholic.ac.kr

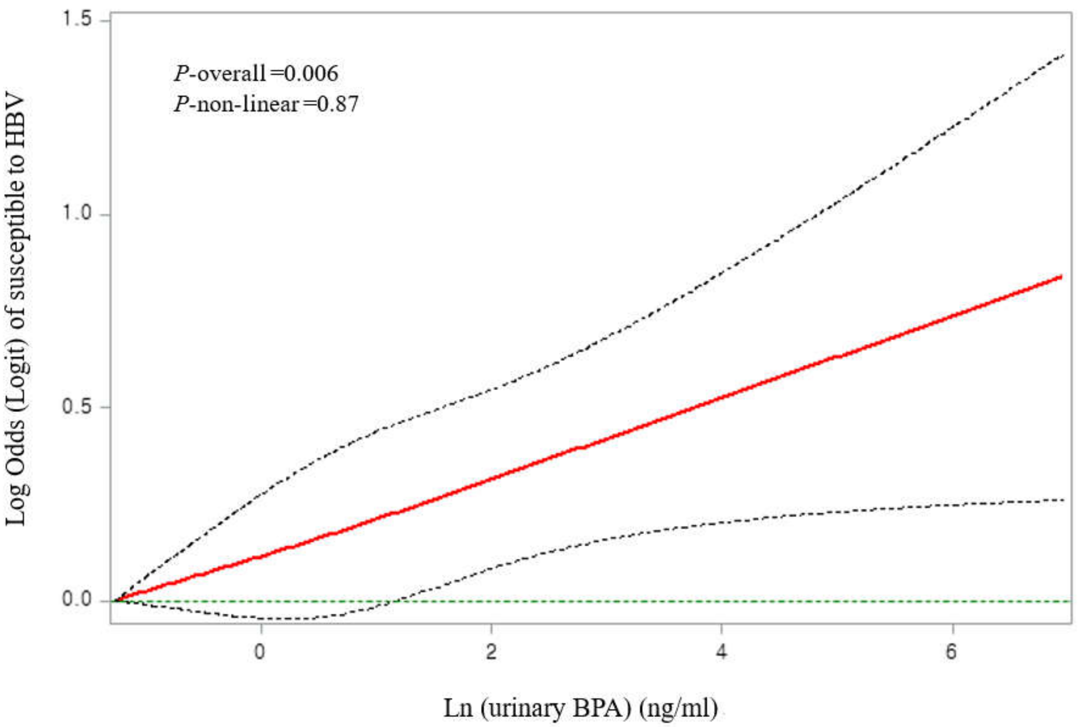

Figure S1. Log-odds of immune response to vaccination against hepatitis B virus according to natural log-transformed urinary bisphenol A, adjusted for natural log-transformed urinary creatinine, survey cycle, age, sex, race/ethnicity, country of birth, household income, body mass index, and smoking status. The solid line represents the smoothing trends estimated from the restricted cubic spline with four degrees of freedom (knots at 10th, 50th, and 90th percentiles), and the dashed lines represent its 95% confidence intervals (CIs).

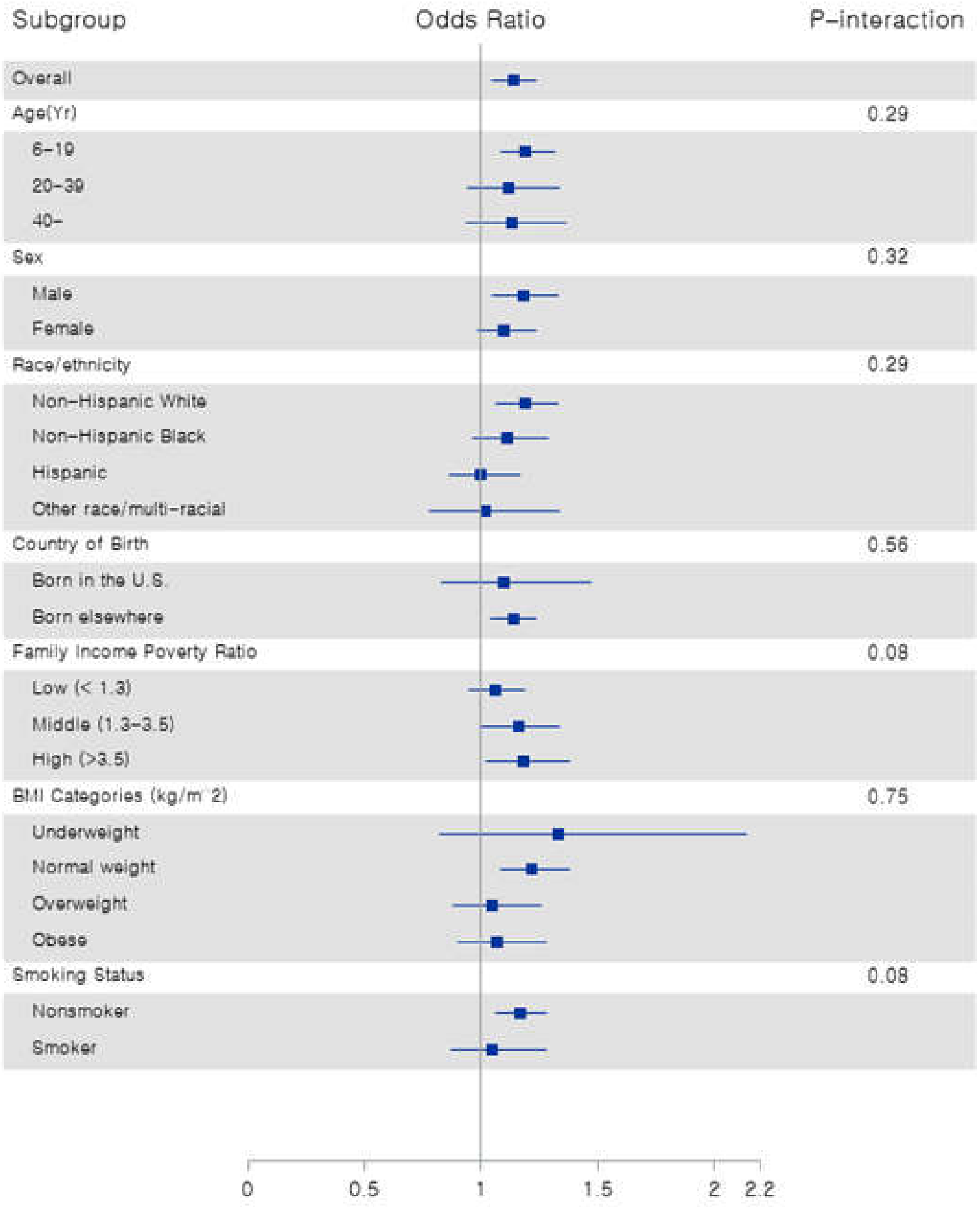

Figure S2. Subgroup analysis of odds ratio (OR) of susceptibility to HBV according to urinary BPA level. All covariates included in model 3 were adjusted.
